# Community engagement approaches to improve health: a cross-case study analysis of barriers and facilitators in UK practice

**DOI:** 10.1186/s12889-025-21902-5

**Published:** 2025-02-24

**Authors:** Anne-Marie Bagnall, Jane South, Karina Kinsella, Joanne Trigwell, Kevin Sheridan, Angela Harden

**Affiliations:** 1 School of Health, Centre for Health Promotion Research, Leeds Beckett University, Leeds, LS1 3HE UK; 2 School of Health, Leeds Beckett University, Leeds, UK; 3 (Formerly at) Institute for Health and Human Development, University of East London, London, UK; 4 Consultant in Community Engagement and Community Development: Implementation, Strategies, Training and Research, London, UK; 5 School of Health Sciences, City University, London, UK

**Keywords:** Community engagement, Health and wellbeing, Cross-case analysis, Health equity, Health inequalities, Public health, Health promotion, Qualitative research

## Abstract

**Background:**

Interventions that engage communities have been shown to improve health and wellbeing in disadvantaged groups internationally, but there is little evidence on current community-led practice, particularly in relation to the process of community engagement. This paper presents a qualitative cross-case analysis of barriers & facilitators in six UK community engagement projects, using different models of community engagement.

**Methods:**

The primary sampling criteria was the type of approach to community engagement, using a conceptual framework with four main groups: Strengthening communities; Volunteer and peer roles; Collaborations and partnerships; Connecting to community resources. Qualitative interview-based methods (semi-structured interviews and focus groups) explored community and professional perspectives in depth. Thematic analysis was used to analyse the data, building within-case studies before comparing findings and using an iterative process to build explanations in a cross-case analysis.

**Results:**

Fifty-five people (28 community stakeholders and 27 professional stakeholders) from six selected case study projects took part in the research. Key themes related to successful community engagement were: trust within the community and between community members and service providers; respect for community members’ expertise; allowing sufficient time for relationships to establish and for outcomes to be seen; commitment of key people; and flexibility.

**Conclusions:**

This qualitative case study research found that in successful community engagement projects, community expertise is respected and valued, allowing community members to be fully involved and take ownership of the projects. Sufficient time should be allowed for this process. Flexibility and adaptation of project materials, protocols and role descriptions is important in overcoming barriers to community engagement.

## Background

Community engagement has been defined as the ‘direct or indirect process of involving communities in decision making and/or in the planning, design, governance and delivery of services, using methods of consultation, collaboration, and/or community control’ [1]. It is an ‘umbrella’ term, covering a wide range of community-centred approaches (such as community development, peer support, or time banking) with differing populations, intentions and levels of engagement [2]. Recent attempts to categorise these approaches have recognised that there is no ‘one size fits all’ best approach, and that the most effective projects may draw on a range of approaches to create a ‘bespoke’ design that responds to the needs and assets of the community in context [3, 4] .

Community engagement for health is defined in UK national guidance [5] as being about people improving their health and wellbeing by helping to develop, deliver and use local services. It is also about being involved in the local political process. Community engagement can involve varying degrees of participation and control: for example, giving views on a local health issue, jointly delivering services with public service providers (co-production) and completely controlling services. In the UK guidance [5], community engagement includes activities to ensure that community representatives are involved in developing, delivering or managing services to promote, maintain or protect the community’s health and wellbeing. These activities can take place in a range of settings, including care or private homes, community or faith centres, public spaces, cyberspace, leisure centres, schools and colleges and children’s centres. Named community engagement roles include (but are not limited to) community champions, community or neighbourhood committees or forums; community lay or peer leaders [6].

Previous research has found that the more a community is supported to take control of activities to improve their lives, the more likely it is that their health will improve [7]. A systematic review of 319 studies of different types of community engagement for health inequalities concluded that community engagement interventions “are effective in improving health behaviours, health consequences, participant self-efficacy and perceived social support for disadvantaged groups” [1].

Community engagement for health can involve varying degrees of participation and control, from giving views on a local health issue, jointly designing and/ or delivering services equally with public service providers, to communities completely controlling services [8].

A conceptual framework of community-centred approaches, developed by Public Health England in tandem with the NICE guidance that this research informed [3, 9], presents four main approaches:

- *Strengthening communities* - approaches build on community capacities to take action together on health and the social determinants of health;
- *Volunteer and peer roles* - approaches enhance individuals’ capabilities to provide advice, information and support or organise activities around health and wellbeing in their or other communities;
- *Collaborations and partnerships* - approaches involve communities and local services working together at any stage of the planning cycle, from identifying needs through to implementation and evaluation (includes co-production);
- *Connecting to community resources* - approaches connect people to community resources, practical help, group activities and volunteering opportunities to meet health needs and increase social participation.

While the ‘Strengthening Communities’ approach seems to suggest the highest levels of community control and community power, in practice there can be high levels of community control in any of these approaches, and they are often combined.

In terms of differential impacts of these approaches, a large systematic review found that peer-delivered interventions had the highest effect sizes in terms of reducing health inequalities, possibly because of their narrower focus, while “community empowerment models might be expected to have smaller effects over a broader range of health and social outcomes” [1]. Later research found that higher levels of community engagement (i.e., being involved in the design, delivery and evaluation of a project) may be associated with better health outcomes [10, 11]. However, a systematic mapping review conducted as part of the UK guidance update [12], which included descriptive reports of community engagement practice as well as research and evaluation, found that activities with higher levels of community engagement were least likely to be published in journal articles or evaluation reports. This lack of evidence on implementation of the types of community engagement activities which may have the greatest potential to reduce health inequalities represents an evidence gap that this qualitative primary research project seeks to address.

While the concept of patient and public involvement and engagement (PPIE) is becoming more prominent in research, which is a welcome development, community engagement in the context of this research project, and the NICE guidance [5], refers primarily to community involvement in the design and delivery of health-related activities in their own communities. There is potential overlap with PPIE if there is also community involvement in research or evaluation of these activities, but they are not the same. The formal structures surrounding the process of researcher-led PPIE as a transactional instrumental strategy [13, 14] currently do little to address the power imbalances and other barriers to participation faced by disadvantaged and marginalised population groups (with the exception of community-based participatory research), whereas community engagement as a community-centred approach to health is associated with increased empowerment of individuals and collective community control via a number of pathways that support the involvement and inclusion of marginalised groups, thus improving their skills, knowledge and connections as well as their health [15].

Previous UK guidance reported many potential barriers to community engagement [16] - such as lack of infrastructure to encourage multisector collaboration, dominance of professional cultures and ideologies, capacity, willingness and skills of community and professional stakeholders to get involved - and therefore the updated UK guidance [5] focused on what helps or hinders community engagement in the UK. The related systematic review of barriers and facilitators to community engagement categorised these into contextual (or pre-existing) and process-related barriers and facilitators. The systematic review also noted a lack of studies which attempt to evaluate how to overcome identified barriers to community engagement [17].

Based on these previous research findings, and a conceptual paper [18], we anticipated evidence gaps on empowerment approaches and unexpected effects. We therefore aimed to include as case studies at least one community-led initiative for which there was no substantial evaluation report (as a proxy indicator of less ‘professional’ involvement), and at least one project for which implementation, delivery or impacts had not gone to plan.

This paper reports the findings from qualitative interviews and focus group discussions in a cross-case analysis of six case studies of approaches to community engagement to tackle health inequalities in England, with an emphasis on common influencing factors that facilitated and overcame barriers to community engagement in practice [19].

The six case study sites were from a range of geographical locations in England and represented a range of approaches to community engagement (see Table 1).

**Table 1:** Case study sites

Project	Approach to Community Engagement	Population	Type of Activity	Brief description
**Leeds-** Gypsy and Traveller Exchange (GATE)	Strengthening communities	Gypsies and Travellers	Community Health Needs Assessment (CHNA)	Leeds Gypsy and Traveller Exchange (GATE) is a registered company, a charity and a community members association which is led by, and representative of, Gypsies and Irish Travellers [20]. The overall aim of Leeds GATE is to improve the quality of life for Gypsy and Irish Travelling people living in or resorting to Leeds
**Dudley-** ‘Life is Precious’	Volunteer & peer roles	Local Black, Asian and Minority Ethnic (BAME) people	Community health champions; art workshops; focus on cancer	‘*Life is Precious*’ is a cancer health improvement project commissioned by Dudley Public Health Community Health Improvement Team [21]. The project used a creative arts approach to engage local people from minority ethnic communities in a dialogue around cancer
**Wandsworth-** Church-based Family Therapy	Connecting to community resources; partnerships/ coalitions	Local people; BAME; faith; mental health	Co-production model, empower pastors to disseminate key messages around relationship building and mental health to the local community	The *‘Church based family therapy in Wandsworth’* project, is a partnership between Wandsworth NHS clinical commissioning group, South West London Mental Health NHS Trust, Wandsworth Community Empowerment Network (WCEN) and the Pastors Network for Family Care [22]. The aim of the project is to increase uptake of early intervention and decrease use of acute mental health services among the BAME community and to embed therapeutic skills inside communities
**Liverpool-** Friends of Everton Park (Natural Choices)	Strengthening communities; volunteer and peer roles	Local community in a deprived area	Regeneration; capacity building; volunteers; health and wellbeing; natural environment/ green space	The Friends of Everton Park are an open voluntary organisation of partners and communities who were established in 2010, to work together to make the Park a common treasury for all. The Friends run an annual programme of events where the local residents engage into music, sports, arts and leisure events. In 2012 the Natural Choices Programme was delivered, funded by Liverpool Primary Care Trust (PCT) and ran in partnership with The Mersey Forest. The aim of the programme was “to promote health and wellbeing in Liverpool residents by utilising natural environments and the talents and interests of communities” [19]. A total of 38 community projects were funded by the Natural Choices Programme, including the development of the Faith plot by Friends of Everton Park
**London-**Youth.com	Strengthening communities; partnership/ coalitions; peer roles	Young people	Developing partnerships and community projects; health promotion; changing attitudes	Youth.com (originally called Youth.comUnity) was created by the Well London Alliance to be led by Central YMCA (CYMCA) [19]. Two Youth.com Programme coordinators, each in 10 of the target sites, aimed to recruit two Young Ambassadors in each site, provide them with some expenses, project money, training, and support, and network them together and with other external youth organisations. The Young Ambassadors would then set about engaging with other young people from the target areas and signpost them into the various activities as well as using the small project funds to create their own activities
**Margate-** Connecting Communities (C2)	Strengthening communities; partnership/ coalitions	Local people in deprived areas	Supports delivery of a 2-year intervention designed to improve the health and wellbeing of deprived communities by setting up a 'People and Services' Partnership, led by local residents supported by a multi-agency team of service providers	Connecting Communities (C2) is a framework for transformative change in disadvantaged communities, based on evidence of what works from experience and reflective practice. It is a seven-step process that engages both service providers and residents. It was implemented in two Kent neighbourhoods – Cliftonville West in Margate and Newington in Ramsgate from 2012

The following research questions guided the study:

1. What barriers and facilitators affect the delivery of effective community engagement activities – particularly to people from disadvantaged groups?
2. How can the barriers and challenges be overcome?

## Methods

A multiple case study design was used, with the projects as the cases [23]. Qualitative methodology was used to gain in-depth understanding of the community engagement processes operating within the specific social contexts of each case [24] and to retain flexibility to pursue lines of investigation [25, 26]. A holistic, multi-dimensional view of each community engagement project was first built through fieldwork and analysis, examining retrospectively the journey of each project from development, through to delivery and evaluation. The final stage was cross-case analysis to build explanations through rigorous qualitative techniques.

### Sample selection

An online Register of Interest, established as part of the systematic mapping review [12] carried out to inform the UK guidance [5] identified relevant English projects willing to be a case study. The Register was built from a Call for Evidence that was distributed by the National Institute for Health and Care Excellence, and by academics at both contributing institutions, using networks of national, regional and local voluntary and community sector organisations that they were already in contact with through previous research. 78 organisations responded to the Call for Evidence, of which 41 projects met the eligibility criteria for the mapping review. Purposive sampling was used to select six community engagement projects from these 41 as case study sites. The primary sampling criteria was the type of approach, using the four main groups of the ‘Family of Community-Centred approaches’ [3, 9] as a conceptual framework: Strengthening communities; Volunteer and peer roles*;* Collaborations and partnerships; Connecting to community resources.

Secondary sampling criteria, used to gain maximum variation in the sample [26], were:

- Population group;
- Definition of community: geographical, cultural, common interest or other definition;
- Geographical location (spread in England particularly between North and South);
- Type of activity (e.g. community health champions; community development; volunteering)

Projects that volunteered to be case study sites and were eligible were mapped against the sampling criteria and an initial ‘long list’ of potential projects drawn up. The final six sites were selected at a consensus meeting of the whole research team (all authors), where each was judged in terms of fit with the criteria and the need to ensure a broad sample.

In collaboration with the community project leads, who acted as gatekeepers to recruitment, a sample of participants was identified within each case to encompass different roles and responsibilities. This included public health commissioners, project managers, practitioners, project staff, representatives from partner organisations and community stakeholders. We aimed to give two weeks between first mention of the project to participants and contact from the research team.

### Data collection

Qualitative semi-structured interview-based methods were used to explore community and professional perspectives in depth [27]. Focus groups were used where community participants usually met as groups [28, 29]. The research team were invited by project staff to attend groups to carry out the focus group discussions at the end of planned activities. This followed discussion of the research by the project lead with group members, who were given the project information sheet and consent forms before deciding whether to take part. All data collection took place in-person between September 2014 and March 2015.

The choice of methods was underpinned by wishing to retain where possible a naturalistic approach to data collection [30], fitting with the way each project operated and also supporting the preferences of participants on how the interview was conducted. In most cases individual interviews were conducted with stakeholders, but in some instances two participants chose to be interviewed together in a dyad [31]. This was usually where people routinely worked or volunteered together.

The main topics explored in interviews were:

- Project activities, purpose, and background;
- Community involvement in design, delivery and evaluation of project; nature and extent of involvement; barriers and facilitators;
- Community members’ impact on decisions;
- Whether community members feel accepted and included in the project;
- Benefits to community members, wider community and wider impact of project;
- Unanticipated effects and drawbacks;
- Connections with other projects in the community.

### Analysis

Thematic analysis was chosen to analyse the qualitative data [32, 33]. The approach was broadly inductive to ensure all relevant themes were mapped, but informed by the overarching research objectives. In the first stage, an initial coding framework was developed to encompass themes emerging from the interviews and topics of interest identified from the research questions, the logic model in the scope provided by commissioners, and the conceptual framework developed in the earlier systematic review [1]. Transcribed data from the first two case studies was coded by two reviewers working independently (JT,KK). The review team then met to agree a common framework and initial thematic categories. The thematic framework was expanded and refined as analysis continued, until all the themes were coded and organised into subcategories within the whole data set.

Explanations were built within-case through the production of individual case study reports for each project [23], which organised and displayed the data by theme with quotations in a standardised format to allow for later cross-case analysis [33]. The reports also included narrative summaries of project context, history and networks drawn from interviews and project documentation where available. Each report (with identifying details removed) was checked for authenticity by the project leads or other appropriate stakeholder for that project.

Cross-case analysis involved comparing findings and using an iterative process to build explanations [23]. A matrix was produced as a visually ordered display to represent the whole data set and summarise the themes across each case study [33]. The matrix was used alongside the case study reports to produce a narrative synthesis across the main themes, drawing out cross-cutting themes and returning to the data as necessary to build explanations. All researchers involved in the data collection were involved in checking the final narrative account.

## Results

Fifty-five people (28 community stakeholders and 27 professional stakeholders) took part in five focus groups and 26 interviews across the six sites (see Table 2).

**Table 2:** Case study participants

Case study	N of interviews	N of focus groups (n participants)	N professional stakeholders	N community stakeholders	Total N participants
Leeds GATE	3^a^ (4P)	1 (3C)	4	3	7
Life is Precious	5^a^ (6P)	2 (10C)	6	10	16
Wandsworth Church-based family therapy	6P	1 (10C)	6	10	16
Friends of Everton Park	2C	1 (3P)	3	2	5
Youth.com	5 (4P, 1C)	0	4	1	5
Connecting Communities	5^b^ (4P, 2C)	0	4	2	6
**TOTAL**	**26**	**5**	**27**	**28**	**55**

(P) Professional stakeholder; (C) Community stakeholder

^a^One interview was conducted as a dyad (with two participants)

^b^One of the interviewees was both a professional stakeholder and a resident

Themes from the cross-case analysis relating to barriers and facilitators to community engagement (see Table 3) were organized into a loose reporting framework using categories from the related systematic review [17] of *contextual* (or pre-existing) barriers and facilitators, and direct influencing factors on the community engagement *process*..

**Table 3:** Cross-case analysis matrix

	**Life is Precious**	**Leeds GATE**	**Wandsworth church-based family therapy**	**Friends of Everton Park**	**Connecting communities**	**Youth.com**
Need for project	Cervical Monologues- previous awareness raising project. 2007 cancer reform strategy highlighted a lack of awareness of cancer in BAME communities.	Census data did not include Gypsies and Travellers as an ethnic minority until 2011. General poor health of the Gypsy & Traveller population was well known.	Over representation of BAME population accessing urgent mental health care services. High profile case – church member’s brother died “in the process of being restrained by police”.	Develop capacity/ increase social capital within deprived communities. Explore new ways of engaging deprived ‘vulnerable’ communities in health. Funded projects where health needs were greatest in the city and green space.	Social housing estate suffering from multiple deprivation, poverty, unemployment, poor housing, crime and anti-social behaviour, including knifings and substance misuse. This in turn led to fear, isolation, and desperation. An incident in the mid-90 s involving a Molotov cocktail was described as a “tipping point”.	
Barriers to CE	Formal DBS checks & paper work- therefore training was informal. Running workshops during religious group times, timing is key- e.g. not when children need picking up from school. Worries around discussion of ‘sensitive’ topic in the community.	Attitude of professionals (mainly GP receptionists) led to negative experiences for community members. The CHNA could have been promoted more within the community prior to being undertaken. Lack of support and direction from a professionally led steering group. Lack of funding, limited training, limited resources available for delivery, lack of childcare facilities.	Funding challenges/ barriers to overcome to get funding. Stigma associated with mental health—Stigma was considered a barrier in regards to engaging community members in the delivery of the project as well as challenging the perceptions of the wider community. Therefore, it was recognised education surrounding mental health was needed prior to engagement in the project. Conflict of ideologies regarding the origins and treatment of mental health- Personal beliefs were considered a potential barrier to engagement. Resistance from community members to work in partnership with mental health services. This resistance also related to a distrust of the mental health service and that involvement in the project would lead to a more *“secular kind of way of doing ministry.”* Maintaining credibility as a religious leader was considered a potential concern surrounding involvement. Undertaking the training—community members described as often intellectually challenging and ‘daunting’. Time and commitment was discussed as a barrier to engagement in regards to attending meetings and the training, completing assignments and supporting the wider community. A lack of financial compensation for time.	Weather – “wettest year on record bar one”. Time: short term funding – project funding was only one year. Community members’ time – recognising volunteers have other commitments. School built on plot—plot decreased in size. Paperwork – time spent completing funding applications and completing the evaluation.	History of poor relations between service providers and residents—makes residents cynical and sceptical and often unwilling to engage because they find it difficult to believe anything is going to change. A lack of time. The timing of meetings could be a barrier to genuine engagement. Meetings held in the day excluded those that work, and meetings held in the evening or weekends were often resisted by service providers.	Lack of time to build relationships. Insufficient funding. Not having a clear plan in place from the start led to delayed implementation. Attitude of partnering organisation in Well London towards young people (well-meaning but no mechanism for inclusion/ or not taken seriously). Just building a team of peer trainers from cohort who passed through & then money ran out—so sustainability (lack of renewed funding) a problem.
Facilitators to CE	Culturally appropriate and accessible training and resources –running same sex groups where appropriate. On-going conversation about acceptability images & messages that could be fed back to communities. Venues that people are familiar with. Community representative in each group so if people did not want to approach the professional they could ask their CR. Sensitive issues- arts was a good way to engage people in a safe environment. Identifying target audience and languages for interpretation purposes. Community members led the design and delivery – consultation with community members. Engagement in evaluation from the start- survey best time to run workshops, barriers, preferred type of art, childcare, food, etc. Reflections at the end of each session to assess what did/did not work well.	Strong, supportive reference group of two members of the community and a GATE worker. Dedicated input from a well-respected community midwife. Resource group were able to take ownership of the project from the start and do their own promotion and design delivery materials. Training.	Co-production through the involvement of, and developing relationship with, key organisations (e.g. the NHS Trust). Ownership surrounding decisions made relating to the design and delivery of the project, and how it would move forward in the future. The role of the Wandsworth Community Empowerment Network, in bringing individuals and organisations together as well as mediating & negotiating the relationships. Time was needed to establish these relationships and networks for the community engagement project to be successful. The influence of key and respected individuals. Organisational and individual commitment/ responsibility to make positive societal changes. Recognising Pastors are of value, and have useful skills and experience, in making these positive changes (recognising social capital). Increasing the personal assets of Pastors via training and ensuring they were supported during the project (training and project delivery). Ensuring the training was adapted to meet the cultural needs of community members.	A network of support (collaboration) – from commissioners/ external project managers as well as other funded projects. Network of support increased via the use of events bringing all 38 funded projects together. At this events projects shared ideas and swapped resources (e.g. left over compost/ timber) to assist other projects. Trust in staff overseeing the delivery of the programme (Natural Choices) – consistency in staff important (project took place during change over from PCT to CCG). Passion of community members to make a difference. Community were given ownership of decisions made surrounding the design and delivery of the project. Flexibility in project delivery – protocol could change during the funding period to meet needs of the community. Having various roles volunteers can assist with to meet their own interests. Funding –simplified funding application to increase number of funding applications from community led organisations. Trust in key individuals.	Having a receptive attitude to change and to the need for resident-led action. Enabling a community voice. Listening by service providers and the perception of residents of being genuinely listened to. Having a strong but flexible evidential methodology for community engagement. Giving time for things to work. Having sufficient funding to enable the community engagement. Having strong mechanisms for support and shared learning that enable and encourage residents to achieve their own goals. Having good communications channels and media in place was seen as an essential facilitator by the local stakeholders. Communications needed to be adapted to audience, new media for some, older style letters and newsletters for others, but most of all word of mouth. Using a personal invite to residents to take part in the engagement process. Incentives in the form of a raffle with prizes donated by local businesses. Having meetings at convenient times. Having the right venue for events and meetings. Having childcare or activities available to engage children. Providing a social atmosphere at the community engagement events and meetings. Feedback and feeding back quickly was seen as important by all respondents. Providing materials in different languages where appropriate. Keeping the momentum going. “Quick wins”.	Supportive attitude of local voluntary/ community positive & helpful. Flexibility of developed model to adapt to local conditions. Putting right support team (right skills in right places).
Training	Flexible resources. Acknowledge skills community members bring: “*It was very much designed to be flexible & to enable them to do really as little or as much as they wanted to do*”	Full week of training; confidence building, public speaking, orientation work. Flexible training that suited the delivery needs of community members- able to travel daily to the course.	Two year accredited training course – considered a facilitator to CE and benefit to community members. Adapted to meet the cultural needs of the group. Support during training – enabler to community engagement. Barrier – time to attend training and complete assignments.	Informal training, wood cutting, knowledge building around planting, types of greenery etc.		
Benefits for community members	Confidence building, social aspects- mixed with people from different cultures and religions, gained knowledge, empowerment. Badges to highlight their role.	Training. Gaining respect from other community members. Social capital, peer support.	Personal development – accredited qualifications and increased knowledge/ awareness and skills. Greater participation in civic life/ empowered. Aided personal life/ role as Pastor.	Increase in confidence as project grew. Pride in project. Use of green space for own interests (e.g. growing vegetables/ plants). Increase in skills and knowledge – capacity building.	All respondents were clear that the C2 framework had benefitted residents’ personal growth and sense of purpose: *“Yeah. Yeah, there's one individual who wouldn't come into a meeting, he wouldn't even say hello if you walked passed him. Now he's quite happy to stand up and speak at a meeting in front of everyone. Completely different person. He's actually done presentations for different groups as well.” (Community Stakeholder)*	Very positive results for young ambassadors—many went on to employment, university etc. and had new skills.
Acceptability of project	Senior buy-in from business case-key factor to success. Strong senior leadership within team.	No buy-in from steering group. Mixed responses from GP surgeries-which acted as gatekeepers and possibly had an impact on take-up in their area.	Overall considered a success. Viewed as an acceptable ‘model’ of community engagement. Pastors have sustained engagement (moved to Y2 training)/ commitment. Recognising the importance of utilising community infrastructure – despite concerns surrounding the trust working with religious organisations. Uptake among other groups. Pastoral role and systemic therapy considered complementary.	Described as an enjoyable project. Number of volunteers increased over project period.		
Perceived impact on community participants	Perceived wider awareness, “*less of a taboo*”. Behaviour change- more likely to be screened. ‘*don’t throw away tester kits anymore’.*	Raised awareness of individual health issues within the community. The resource group were trusted and respected within the community. Some community members opened up and shared their stories.	Believed to help normalise discussions surrounding mental health. Aided clinicians understanding of cultural issues related to mental health. Increase of BAME IAPT service users. Number of individuals attending new groups to seek help from Pastors.	Improved green space for the wider community to utilise. Increased interest in growing plants/ vegetables within the community. Site for local schools to use. Increased emphasis on using local green facilities – e.g. allotment and Everton Park. Increase in physical activity levels and well-being.	New relationships with services; benefits to residents, service providers and staff. New relationships in community. Community members expressed a sense of feeling safer due to the newer sense of community.	
Linked work-development of new projects	3CHCs as a direct result of project. New project for taxi drivers increasing health awareness. Predicted that more people will cancer screen now and attend smears etc. Some CHCs are able to deliver training in their communities. Breast cancer awareness event hosted by community members.	Advocated towards creating improved services for G&T community. Creates a strategic plan and commissioning strategy with Leeds West CCG.	Imams to participate in Y1 of training. New activities in churches e.g. Family Time and Monday Night Life to provide families with further support. Ideas to launch ‘The Black Barbers Project’ – provide Barbers with information to signpost clients to mental health services. Developed/ strengthened relationships with other networks/ organisations. Development of a website promoting the project (for one church).	Commissioners used learning from the project to develop further engagement work based on the model used. Heritage trail – lottery funded programme. Won Kew Gardens Grow Wild award in conjunction with Manchester. Increase in PA projects. Developed connections with other community groups through Natural Choices.	The partnerships are well established now and the process is showing signs of sustainability. New people are joining the partnership committees, and residents are meeting with other C2 sites, and applying for funds Many new and emerging projects e.g. Waste Forum; Recycling projects; Gardening Clubs; Asset Mapping; Youth groups; play park; new community wood.	
Unforeseen issues	Art work displayed in hospitals. Real sense of pride and ownership.	Due to bereavement, the resource team stopped working on the project and an independent freelance worker completed the CHNA. Potential for loss of ownership of end product.	Greater interest than expected. Negative impact on pastors in terms of time spent on training and delivery.	Greater interest than expected.		

### Contextual barriers

Contextual barriers to community engagement were stigma, role conflicts, funding and cultural barriers.

Stigma was a common perceived barrier in five of the six projects, both within the community, and in attitudes from those outside the community. Some concerns centred around “sensitive” health issues, such as mental health or certain types of cancer, with reluctance to discuss these issues, and difficulties in dealing with them, such as not attending cervical screening appointments. Other concerns were unhelpful attitudes of professional staff towards marginalised communities:

*“… in these communities, [there is] a lot of misunderstanding of what is mental health and mental illness; a lot of fear, a lot of stigma about mental illness. So when you had people who were coming from that mind-set, there was actually quite a number of steps and quite a lot of engagement and quite a lot of learning that was about paradigm shifting that needed to happen before you could even get engagement.”* (Professional Stakeholder, WCEN)

Participants in the Youth.com project reported that the attitude towards young people was that it was great to have them involved as participants, but that they weren’t capable of influencing decisions in a useful way. Some felt that they were being kept away from power, and others that it was a question of respect from adults for young people, presenting a barrier to full engagement.

In the Leeds GATE project, the unhelpful attitude of some professionals resulted in negative experiences for community members when they were asked to display posters in clinic and GP practices:

*“So some people would include the information and say that’s not a problem yeah we’ll put it on the board, others were really offensive and made them feel very small.”* (Community Stakeholder, Leeds GATE)

In one case study on neighbourhood regeneration, a history of poor relations between service providers and community members was seen as a barrier to engagement, making residents cynical and often unwilling to engage because they found it difficult to believe anything will change:

*“To start with – residents have been consulted to death, you know, and, "Do you know what? We would like another bin," and they'd get a bench (laughs) because that's what the service providers have said that you [need]…Yeah. So they just think, well, what's the point of saying anything? It's like, you know, you're not going to listen to me anyway.”* (Community Stakeholder, C2)

#### Role conflicts

Some projects reported perceived role conflicts for community or professional stakeholders as a barrier to community engagement. In the Wandsworth Church-based family therapy project, this related to concerns among some community Pastors due to historical *“problematic”* associations between faith and mental health structures:

*“There needs to be some level of interest in the faith groups for wanting to do this. Historically, faith and mental health is a problematic, and not very easy, association. So [people with mental health problems], clinicians, practitioners in mental health services were often very, very suspicious of faith leaders, and faith leaders were suspicious of the mental health structures.”* (Professional Stakeholder, WCEN)

#### Funding

Lack of funding and complicated application processes could be both a contextual and a process barrier to community engagement. Lack of funding led to limited opportunities for training, limited resources for project delivery and lack of childcare facilities and other necessary resources to support engagement of community stakeholders. Short-term funding led to a need to gain further funding, which some community groups felt ill-prepared for in comparison to more established groups competing for the same funding:

*“And also sometimes the governance of community organisations is not always as robust. And so if you're going for a bid and, you know, […] bids for it, they will fill in the form perfectly, they have an audit committee, they'll have a Director of Finance, they'll have a whole system behind them which kind of makes sure they're able to kind of fill the requirements of a funding organisation, whilst community groups don't have that, which puts them at a massive disadvantage…”* (Professional Stakeholder, WCEN)

In Youth.com, the original funding amount was halved, which reduced staff capacity and resulted in changes to the model, timing and length of delivery:

*“…our early estimate of what would be ideal would be one coordinator per borough proved in hindsight to be – certainly two across all twenty was nothing like enough, and they (the Youth.com coordinators) put a huge amount of time and energy into supporting the young people.”* (Professional Stakeholder, Youth.com)

Lack of awareness of the purpose, models used and long-term nature of community engagement by wider public health and health services, were sometimes perceived to present a barrier to commissioning, especially of projects which focus on capacity building, co-production and long-term outcomes, but lack immediate health impacts:

“*We’re not perceived to have assets. We’re not perceived to be contributors. We’re not perceived to be actual suppliers of a service, and I think that has to be, again, a shift in terms of can community leaders be trusted to be able to be competent deliverers of this service?*” (Community Stakeholder, WCEN)

Cultural barriers (e.g. religion, gender, language) were significant if not handled correctly. For example, professional stakeholders in one project mentioned that running workshops during religious meeting times had resulted in poor attendance in previous projects. One of the projects needed to put on separate groups for men and women, and took steps to overcome language barriers by using a translating service to provide interpreters:

*“What we were looking for was somebody that could speak the same dialect, and we also explained that we don’t want somebody to just sit there, they have to be engaged in the process and they all vetted for those skills and they were asked to attend a briefing meeting so that they were aware of the … messages.*” (Professional Stakeholder, Life is Precious)

### Contextual facilitators

Contextual facilitators to community engagement were established relationships of trust, enthusiasm of both professional and community stakeholders., and respect from professionals for community knowledge.

Having a strongly established community or network, and trust within that community enabled new initiatives to engage with the community more easily. For example, the role of the Wandsworth Church-based family therapy project in bringing individuals and organisations together was felt to be key. Trust in key individuals was felt to be important, which in one project meant consistency in staffing:

*“What has made it work I think has been the mediation of [organisation]. I don't think it would have worked without a third between the Trust – even though we were a very small department, but I think [NAME] has done a really fantastic job of negotiating across the CCG, the Trust, […], and that’s a very skilled piece of negotiation.”* (Professional stakeholder, WCEN)

Enthusiasm of community and professional stakeholders for the projects and communities involved was particularly useful for getting people involved initially:

*“I think what makes it work well is the enthusiasm of the [community stakeholders] – that's working well – and that enthusiasm is probably driven by increasing demands and the need for supply.”* (Community Stakeholder, WCEN)

Professional stakeholders having a positive attitude towards community stakeholders’ knowledge of their own experience and issues, and abilities to devise solutions themselves, if with some support, was seen as a facilitator to positive community engagement:

*“…by engaging with the young people, we were able to target and deliver projects that young people really wanted on the estate, you know. So it was very much an empowering process that was very bottom up and not a top down process. And you're able to keep people and engage people in such a programme because their voice and working alongside a professional, they're doing a co-production where they are on an equal setting with the professionals. So it was very, very, very different and had a very positive impact.”* (Professional Stakeholder, Youth.com)

### Process barriers

Process barriers to community engagement were training, bureaucracy, lack of support, lack of time and not feeling involved.

#### Training

Time and skills for training was perceived to be a barrier in two projects, with community members potentially being put off taking part due to the time needed to complete the training and concerns about their own ability to engage with a particular learning style—this was particularly an issue in the Wandsworth project where Pastors undertook a 2 year training course in family therapy:

*“… I think we thought that because it was going to be once a month, we’d be meeting, you know actually coming here and having tutorials and everything, that you know, we could manage that. But then the work in between actually coming together is very, very intense and very time consuming. And if you think one of the barriers is the amount of time that it takes to actually do the coursework, to get your hours in, to do your client logs, you know, to do your reflective logs and all the other things, it is a lot to do….”* (Community Stakeholder, WCEN)

#### Bureaucracy

The time and skills/ experience needed to complete paperwork such as funding applications and evaluations was another perceived barrier. Projects tried to avoid formal Disclosure & Barring Service (DBS) checks and paperwork where possible. Staff in one project proposed to overcome these barriers by designing an informal training package, to encourage community members to share their skills, knowledge and experience.

Lack of support and commitment from key people was another barrier, for example a lack of support and direction from a professionally-led steering group was reported by one project:

*“So we were fighting a bit of a battle already you know and we didn’t have key partners consistently on board with us […] disgusted – that nobody turned up at the steering meeting you know.”* (Community Stakeholder, Leeds GATE)

Lack of time was a barrier to attending meetings, promoting initiatives and enabling positive change. This was overcome in one project through ongoing consultation that resulted in the flexible and adaptable delivery of the project:

“*No obligation, just to find out a bit more about what might be involved, what you could do, come along. We did some meetings and just talked to them about how they saw the role really ‘cause it was very much designed to be flexible and to enable them to do really as little or as much as they wanted to do; and the sort of support they would need, the sort of training they would be interested in. And again it was all very tailored to each group because obviously they were all quite different and have different kind of ideas.”* (Professional Stakeholder, Life is Precious)

Time was also needed to develop relationships and trust, and to measure meaningful outcomes. Youth.com noted a lack of time to build relationships, which was seen as not just due to the delayed start of the project, but also due to the target-driven nature of these types of initiatives. It was suggested that funders, who are often target-driven themselves, expect projects to be hitting targets almost from the start, but it takes time to build relationships and establish trust, especially amongst communities that have been traditionally excluded.

Professional stakeholders reported that community stakeholders not feeling involved or represented had in the past presented a barrier to engagement. This was overcome by on-going conversation and consultation, ensuring each individual group was represented.

### Process facilitators

Process facilitators to community engagement were time, commitment from key people, having the right people in the right roles, community sense of ownership, cultural adaptation, good communication, using familiar spaces and organisations, respect, flexibility, support to develop, and ‘quick wins’.

#### Time

Community and professional stakeholders mentioned spending a long time building up a project as a facilitating factor, allowing establishment of relationships and links to existing networks.

*“And to get to where we are now has taken about seven/eight/nine years anyway. So I think all of that kind of work needs to be, I suppose, understood and recognised because it's the groundwork to relationships. The trust is a big thing.”* (Professional Stakeholder, WCEN)

*“I would definitely say if you want to do community involvement then extend timeframes, you know it’s not a 12-week process; this is if you truly want to include community members in it you know, because you have to revisit language.”* (Professional Stakeholder, Leeds GATE)

Commitment and involvement from key and respected people and organisations was seen as essential for success by providing expertise, support, endorsement or by actively recruiting community or professional stakeholders to join the project:

*“The specialist health midwife helped us […]; she was really great […] having a key member within the health you know, on board, wholly on board who isn’t dictating the agenda or manipulating what’s happening, like to fit in with their own work...”* (Community Stakeholder, Leeds GATE)

Both community and professional stakeholders saw having the right people in the right roles as important:

“*The people employed to do the jobs have to have the skills, the experience and the knowledge to do a good job. And I suppose the person who's hiring them needs to know what those skills are in order to hire the right person.”* (Professional Stakeholder, Youth.com)

#### Sense of ownership

Projects which successfully engaged the target communities were those in which the community had ownership and led decisions regarding the design, delivery and evaluation of the project, and its future direction. The Wandsworth church-based therapy project mentioned the related need for service providers to be receptive to change and to the need for resident-led action:

*“Yes, and I think they feel an ownership in terms of how it goes forward. They don’t feel that something’s going to be done to them.”* (Professional Stakeholder, WCEN)

*“So everybody knew what we were planning to do all the time because we had community members that were feeding back and an actual curiosity within the community.” (Community Stakeholder, Leeds GATE)*

Cultural adaptation of training and resources was key to engaging the community, including culturally appropriate and accessible training and resources (e.g., running same sex groups where appropriate), identifying target audience and languages for interpretation, and generally being sensitive to religious and cultural beliefs and needs. Projects involved on-going conversations about acceptable images & messages that could be fed back to communities:

“*Quite a lot of them got the faith and their religious aspect, it was ensuring that we’re not running the workshops that are going to clash with those dates as well and ensuring that they are able to pick their children up on time.”* (Professional Stakeholder, Life is Precious)

“*We were rehearsing, we were looking at questions and starting to collate some questions together and then we were going to the community, how would they feel to be asked this.”* (Community Stakeholder, Leeds GATE)

Flexibility was also important, for example in that projects could change to meet the needs of the community; or the range of roles that volunteers could assist with to meet their own interests. Holding meetings and activities at convenient times for community members was mentioned as a facilitating factor in C2, Life is Precious and in Youth.com by professional stakeholders:

*“Definitely we scheduled meetings at times which were convenient to them, in locations which were convenient to them. That was really important.”* (Professional Stakeholder, Youth.com).

#### Communication

Having good communication channels and media in place was seen as an essential facilitator in two projects, for inviting people to take part, ensuring that meetings and activities are advertised and promoted to all the right people, and giving feedback on decisions being taken forward and other outcomes. In Connecting Communities, professional stakeholders mentioned that communication channels needed to be adapted to the audience: social media for some, older style letters and newsletters for others, but most of all word of mouth. Whereas in Youth.com, social media was more important for engaging younger people:

*“I think another important thing was being able to communicate on a range of platforms, especially the social media platforms. So, Facebook was used an awful lot, as well as WhatsApp. So it was being able to communicate and knowing how to engage with the young people.”* (Professional Stakeholder, Youth.com)

Familiarity, trust and “feeling safe” were important themes, with several projects mentioning use of venues and trusted professionals that people were familiar with. One project selected community representatives from an existing community cohesion group consisting of professionals who were working with minoritised ethnic groups. Community representatives were trusted and respected members of the community who were able to support people to join the project, and help to keep them engaged. Community representatives offered a communication route between the project lead and community members- providing a critical bridging role for less confident community members. Where trust was not there initially, mechanisms were put in place to build trust between community members and service providers:

*“What is really important is, it took part, the [name of group] one for example, took part in their community centre, so it was making them quite relaxed, so making a safe environment in the first place.”* (Professional stakeholder, Life is Precious)

Working in partnership with other local organisations was seen by professional stakeholders as a positive facilitator for the Young Ambassadors in Youth.com**:**

*“For example, a representative from the local schools in the area, I believe, gave the young people £250 towards the talent show. We also had NHS Greenwich Public Health who supported the young people with their event, and the local police were very much involved in the events that the young people delivered. Charlton Athletic also was another organisation that was very supportive […].”* (Professional Stakeholder, Youth.com)

Respect from professionals for community expertise and related concepts of working together and of valuing community members were repeated cross-cutting themes. Engaging the community from the start in design and delivery of the project (and ideally of the evaluation), allowing them to lead and take ownership, and continuing those conversations about what is most acceptable and useful seems to be key.

“*… there’s a particular set of beliefs that we are all committed to, and those beliefs are around the value of human beings, of social – if you use the language social capital, that we all have things to contribute… And that these contributions are valuable, and that they’re valued.”* (Professional Stakeholder, WCEN).

#### Support to develop

Training, although mentioned as a potential barrier, could also be a facilitating factor, particularly if it was seen as a means of supporting community members during the project, recognising their value by gaining qualifications and increasing their personal assets, and encouraging them to achieve their own goals. The time taken to attend the training sessions and complete assignments in one project was felt to be a barrier to community engagement, which community members needed support from peers and professional stakeholders to overcome. Flexibility was felt to be important in terms of content (acknowledging the skills that community members bring; adaptation to cultural needs), delivery, time and place.

Support was also seen as important throughout project delivery, as noted by both community and professional stakeholders in Youth.com:

*“If there wasn't any support, then obviously I think it would have been quite chaotic. I don't think the project would have been quite as successful. You know, it wouldn't probably have happened if that was the case […]. It gave me backup in the sense that if I had an issue […] I could easily phone or send an email, and my questions would have been answered straightaway.”* (Community Stakeholder, Youth.com)

Providing feedback quickly, keeping the momentum going and “quick wins” were mentioned as important facilitators in two projects, where the community were initially sceptical that change would be implemented as a result of the community engagement process. These actions were felt to build trust and show community members that you have listened to them and are serious about taking action:

*“It got positive because people, for the first time, felt they’d been listened to, and the results, you’ve got your top ten or you’ve got your ten top themes there. And everyone was like yeah, right I said that.”* (Community Stakeholder, C2)

## Discussion

Although community engagement interventions have been shown to improve health and reduce health inequalities [1, 10], as demonstrated in the recent Covid-19 pandemic, less is known about how to engage with communities successfully [18], or about what works, for whom and in what circumstances. This research aimed to investigate influencing factors on the processes of community engagement to tackle health inequalities in existing UK projects, as part of evidence commissioned to inform the NICE community engagement guidance update [5]. An earlier systematic review [7] found more evidence on barriers than facilitators, while in 2015 the situation was reversed, with a large number of facilitators identified [17]. The later review noted a lack of studies which attempt to evaluate how to overcome identified barriers to community engagement [17]. This gap is addressed to some extent in this paper, as most included case studies involved stakeholders applying their own learning from previous projects where barriers to successful community engagement had been noted. This focus on overcoming barriers to community engagement fit well with the commissioning body’s stated aim to take an asset-based approach to the guidance, drawing and building on existing community strengths and capabilities, rather than using the disempowering language of need [34–37].

All the case study projects were operating in communities at increased risk of poor health – some were open to all people living in or visiting a particular neighbourhood in an area of high deprivation, while others were targeted towards particular ethnic groups or demographics who experienced discrimination or stigma. In these communities, building trust was an essential first step in the process of community engagement. Our research showed that a context of long-standing relationships of trust between community and professionals was a facilitator to community engagement, but in communities where the trust was not already there, it needed to be built. This process of trust-building was consistently reported to take a long time, given the starting point of high socioeconomic deprivation, stigma and sometimes low social cohesion in these communities. A trust typology, although developed for community-based participatory research and looking at trust between academics and communities, rather than community engagement in practice, characterises six stages of trust, from trust deficit, to functional (trust within the context of a specific project), to reflective (trust allowing for mistakes to be discussed and differences resolved) [38]. These stages are not necessarily different in community engagement in practice, although there may be added complexity due to the range of different organisations, roles and agendas involved. The C2 project recommends a seven step process for community development in areas where there is lack of trust between community residents and services, and was subsequently the subject of a paper examining complexity in public health [39, 40].

Reasons for a lack of trust were rooted in a history of power imbalances, leading to stigma, and other negative experiences in these communities. Therefore it is not surprising that alongside trust, empowerment and control was a common underlying concept, with most projects mentioning a sense of ownership from community stakeholders, combined with respect for their knowledge and experience from professional stakeholders, as facilitators for successful community engagement. Other facilitators of the community engagement process that we found in this study, such as flexibility and support for skills development of community members, may also be argued to work because they demonstrate respect for the commitment that community stakeholders are making, alongside the professional stakeholders, which can go some way towards tackling power imbalances and ‘levelling the playing field’. According to Tritter and Mccallum (2006), and Wallerstein (2002), community level involvement, or collective control, is an important lever for change [41, 42], with community members acting together for mutual benefit [43]. A later paper, although focused on community based participatory research in the US, rather than community engagement in practice in the UK, shared transferable knowledge about mechanisms that facilitate equity of power – these include exposing and understanding oppressive historical contexts, building on community strengths, paying attention to language, making space for deliberative dialogues (rather than one way information), creating structures that support equity in collaborations, and supporting shared power [44]. These principles are reflected in successful community engagement projects in this research.

It is important to pay attention to this underlying context of trust and power and to take steps to address these potential contextual barriers by applying the process facilitators identified in our research because, if successful, community engagement projects foster increased self-efficacy, improved health and social support in populations at highest risk of poor health, thus reducing health inequalities [1].

One of many ways towards mutual respect and power sharing in community collaborations is the development of agreed shared language and ways of working. This is a fundamental concept in community-based participatory research, as well as in community engagement in practice [45–47]. Although our research project was not CBPR, the complexity of the research, policy and practice environment surrounding community engagement is partially reflected in our use of language during the project. We had originally used the term ‘community members’ but we changed this to ‘stakeholders’ partly to reflect the feedback of project staff and volunteers, as many of the non-professional community members were volunteers and/ or recipients of community engagement activities – often progressing from recipient to volunteer – and felt that they had as much of a stake, if not more, in the project as the professional stakeholders. The differentiation between professionals as ‘stakeholders’ and community members as non-stakeholders was also felt within the research team and by project staff to perpetuate a perceived power imbalance between professional and ‘lay’ roles which (often incorrectly) infers that it is the ‘professionals’ who have greater knowledge. This does not reflect the way that grassroots community development projects work – related research has shown that co-production with people with lived experience of the community context is an important factor influencing the success of community engagement initiatives in tackling health inequalities [1, 10].

All six case studies were underpinned by elements of different theoretical approaches, including community health champions [48, 49], co-production [50–52], community development, diffusion of innovation theory [53], popular opinion leaders [54]. What they had in common was that they were either community-led from the outset or professional stakeholders actively encouraged and supported community members to take ownership of project design and delivery.

According to South and Phillips (2014), community engagement is a pluralist concept that can include delivery mechanisms, direct interventions, collective action and increasing community influence over the health system [18]. They recommend taking a systems approach to community engagement initiatives and their evaluation that recognizes the complexity of community action and the outcomes of change processes within communities and services. More recently, Public Health England issued guidance on whole communities approaches to health and wellbeing, which conceptualises community-centred approaches within a place-based (e.g. city-wide) system [55].

### Strengths and limitations

Case studies examine social phenomena within real-life contexts and are an appropriate design where there are many variables of interest and where there is an interaction between a phenomenon and the context in which it occurs [23]. In this study, multiple aspects of interest were examined including practitioner and community perspectives; support systems and delivery processes; community engagement approaches and practices; outcomes, effects and sustainability. The choice of design therefore fitted with an ‘ecological systems’ approach to evaluation that shifts focus from viewing the impact of interventions on communities to examining the dynamic relationship between an intervention and community systems [56, 57].

Due to time constraints, we were in some cases unable to give the planned two weeks during the recruitment process between first mention of the project to community members and contact from the research team. We did not meet our own target in all cases of five interviews and one focus group, usually due to time constraints but sometimes due to the small size of the project teams.

We were unable to include any rural case studies, despite this being one of our secondary sampling criteria. This may have been due to lack of time or resources in the rural case study that was initially selected. This means that we cannot be sure whether the process of community engagement is substantially different in rural initiatives.

All the included case studies were able to offer positive examples of community engagement; although we had planned to include at least one case study where the process of community engagement had not gone to plan, none were unsuccessful so we were unable to compare features of successful and unsuccessful community engagement initiatives. However, professional stakeholders did volunteer information about barriers to community engagement that had limited the success of previous initiatives. They had used the learning from these negative experiences to improve the chances of successful community engagement in their current initiatives.

We were not able to speak to any community members that did not wish to engage with the projects, so we do not know what might have encouraged or prevented their engagement.

The case study research did not set out to evaluate the success of the included projects in terms of achieving their health- or wellbeing-related objectives, but rather to explore the process of community engagement, and whether that had been perceived to be successful. Therefore, we cannot offer any further insight into which elements of community engagement might be associated with improvements in health or wellbeing, other than to note that all six projects had success in engaging with the community and also in achieving some health- and wellbeing-related objectives.

Despite these limitations, this research contributes new knowledge to the evidence base on ‘what works’ in terms of practical actions to promote trust and balance power, to support successful community engagement for communities at most risk of poor health in a range of locations and contexts. This knowledge represents the perspectives of both community and professional stakeholders.

### Positionality

The researchers involved in this study were all academics located in cities in the North and the South of England. None of us were members of the communities of interest or neighbourhoods included as case studies, therefore our positionality was as ‘outsiders’. While this may present barriers in accessing the most marginalised communities, our strategy of first reaching out via trusted community organisations and people was designed to overcome these barriers as much as possible within a limited timescale. Researchers attended groups and took part in activities before holding focus groups and interviews with community stakeholders – this strategy was designed to promote trust and rapport, again in a limited timescale.

The research paradigm in this qualitative research study is primarily interpretivist, with some constraints to a purely inductive interpretation, as we sought to construct answers to questions agreed with a national body.

## Conclusions

These case studies in community engagement practice in the UK identified a range of barriers and facilitators to community engagement, and ways in which barriers may be overcome. Key facilitators of successful community engagement were: trust within the community and between community members and service providers; respect for community members’ expertise; allowing sufficient time for relationships to establish and for outcomes to be seen; commitment of key people; and flexibility.

Community engagement initiatives need to work with established communities or networks and trusted key people. If communities are fragmented or trust does not exist between community members and service providers, measures must be put in place to establish that trust, and sufficient time allowed for that process to work. Community members’ expertise should be respected and valued, allowing their views to be heard and acted upon, and for them to be involved in decisions made about design, delivery and evaluation, and to take ownership of the initiatives. This can involve a lengthy process if community members are to be fully involved, so sufficient time and resources should be allowed for this. Flexibility and adaptation of project materials, protocols and role descriptions is important in overcoming barriers to community engagement.

This work did not aim to evaluate the effectiveness of the included case study initiatives in improving the communities’ health and wellbeing. Further research on whether, how and for whom successful community engagement is linked to improved health and wellbeing would be useful. Such research would ideally use participatory methods and be community-led in order to be as inclusive of community members as possible, including those who have not taken part in community engagement projects. Consideration should be given to novel methods of data collection such as arts and photography, and to reducing the burden on community members in terms of time and effort.

## Data Availability

The qualitative datasets generated by this study are not publicly available, as to share full interview transcripts would compromise the anonymity of the research participants. However, detailed within-case analyses for each of the six case studies are publicly available in the full report (Bagnall et al., 2016b) at https://www.nice.org.uk/guidance/ng44/documents/evidence-review-6 or https://eprints.leedsbeckett.ac.uk/id/eprint/2344/ or from the corresponding author on reasonable request.

## Abbreviations

BAME: Black, Asian and Minority Ethnic
C2: Connecting Communities
CCG: Clinical Commissioning Group
DBS: Disclosure & Barring Service
GATE: Gypsy and Traveller Exchange
NHS: National Health Service
NICE: National Institute for Health and Care Excellence
PCT: Primary Care Trust
PHAC: Public Health Advisory Committee
UK: United Kingdom
WCEN: Wandsworth Community Empowerment Network
